# Novel approach for electromyography-controlled prostheses based on facial action

**DOI:** 10.1007/s11517-020-02236-3

**Published:** 2020-08-29

**Authors:** Xiaodong Zhang, Rui Li, Hanzhe Li, Zhufeng Lu, Yong Hu, Ahmad Bala Alhassan

**Affiliations:** 1 School of Mechanical Engineering, Xi’an Jiaotong University, Xi’an, China; 2 Shaanxi Key Laboratory of Intelligent Robot, Xi’an Jiaotong University, Xi’an, China; 3 School of Mechanical and Precision Instrument Engineering, Xi’an University of Technology, Xi’an, China; 4 Department of Orthopaedics & Traumatology, The University of Hong Kong, Hong Kong, China

**Keywords:** Electromyography (EMG), Prosthesis control, Facial action, EMG control model

## Abstract

Individuals with severe tetraplegia frequently require to control their complex assistive devices using body movement with the remaining activity above the neck. Electromyography (EMG) signals from the contractions of facial muscles enable people to produce multiple command signals by conveying information about attempted movements. In this study, a novel EMG-controlled system based on facial actions was developed. The mechanism of different facial actions was processed using an EMG control model. Four asymmetric and symmetry actions were defined to control a two-degree-of-freedom (2-DOF) prosthesis. Both indoor and outdoor experiments were conducted to validate the feasibility of EMG-controlled prostheses based on facial action. The experimental results indicated that the new paradigm presented in this paper yields high performance and efficient control for prosthesis applications.

**Graphical abstract:**
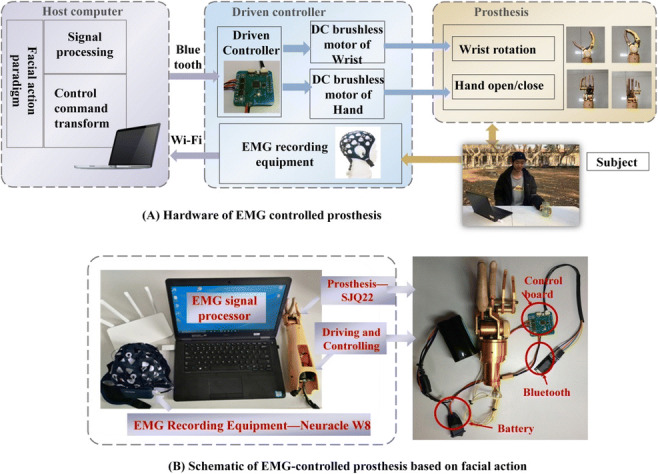
Individuals with severe tetraplegia frequently require to control their complex assistive devices using body movement with the remaining activity above the neck. Electromyography (EMG) signals from the contractions of facial muscles enable people to produce multiple command signals by conveying information about attempted movements. In this study, a novel EMG-controlled system based on facial actions was developed. The mechanism of different facial actions was processed using an EMG control model. Four asymmetric and symmetry actions were defined to control a two-degree-of-freedom (2-DOF) prosthesis. Both indoor and outdoor experiments were conducted to validate the feasibility of EMG-controlled prostheses based on facial action. The experimental results indicated that the new paradigm presented in this paper yields high performance and efficient control for prosthesis applications.

Individuals with severe tetraplegia frequently require to control their complex assistive devices using body movement with the remaining activity above the neck. Electromyography (EMG) signals from the contractions of facial muscles enable people to produce multiple command signals by conveying information about attempted movements. In this study, a novel EMG-controlled system based on facial actions was developed. The mechanism of different facial actions was processed using an EMG control model. Four asymmetric and symmetry actions were defined to control a two-degree-of-freedom (2-DOF) prosthesis. Both indoor and outdoor experiments were conducted to validate the feasibility of EMG-controlled prostheses based on facial action. The experimental results indicated that the new paradigm presented in this paper yields high performance and efficient control for prosthesis applications.

## Introduction

With the growing number of traffic accidents, spinal cord injuries (SCIs), and neurological diseases, approximately 18,496 people lose their hand function every year [9]. Hence, various prostheses-control methods have been developed to aid these individuals to restore some degree of functional ability to interact with their surroundings. Several types of prostheses have been developed, ranging from passive cosmetic prostheses to body-powered limbs, and from electromyography (EMG)-based to electroencephalographic (EEG)-based prostheses [20].

With the development of prosthetic technology, one of the most promising advancement is neural signal control strategies**,** such as EMG and EEG. These are effective system inputs to control prostheses because of their ability to represent a person’s intention [39]. In recent years, several efforts have been conducted to apply brain–computer interface (BCI) technology to prosthesis control [23, 31]. The BCI control uses signals recording from the cortex, which provides direct information related to a person’s intention. Several EEG-based brain-controlled prostheses that utilize various brain activities have been developed, such as steady-state visual evoked potentials (SSVEP) or event-related (de)synchronization (ERD/ERS)-based prostheses [30, 34]. The advantages of brain-controlled strategies can be two-fold. First, they convey information about subject’s intention. Second, they do not rely on a peripheral nerve pathway. However, despite the remarkable success in BCI systems, these approaches often fail to achieve clinical use owing to the instability of invasive EEG signals for long-term applications.

EMG control is widely used as an effective method for assistive prostheses to convey the subject’s intention from muscle contractions [27]. In the 1970s, myoelectric prostheses became significant in rehabilitation and were routinely fitted to upper-limb-deficient clients; clinical evaluations of the functional benefits were conducted [12]. Notably, a real-time myoelectric pattern recognition method was successfully proposed that developed the practical multifunctional prostheses [21]. Hence, EMG-controlled prostheses proved to be some of the most important methods to restore lost limb function [37]. The group of Zhu employed two novel features from discrete Fourier transform and muscle coordination to control upper-limb prosthesis; the classification accuracy was increased by approximately 11% compared with the traditional method in 2015 [15]. To optimize the online performance of EMG-controlled prostheses in real-world scenarios, Al-Angari selected distance and mutual information to discriminate five hand postures with nine different arm positions [2]. In 2017, Li et al. investigated the effect of mobility on decoding limb-motion intentions of amputees and the non-disabled, and proposed the dual-stage sequential method to increase the robustness of multifunctional myoelectric prostheses [38]. In 2019, Michele et al. succeeded in controlling precise artifact hand movements using an EMG linear envelope and muscle activation mapping features, which yielded an online classification accuracy of 91% [7]. Recently, Alexandre presented a real-time gesture recognition system for EMG-controlled prostheses employing an embedded convolutional neural network, and achieved an accuracy of 98.15% [41]. However, most myoelectric prostheses have used the amplitudes of surface EMG signals from residual muscles after amputations, which can only be effective if they satisfy two premises. First, the residual limb muscle can provide sufficient myoelectric signals. Second, the repeated and distinct EMG signal patterns for different motor tasks associated with their limb movements can be activated [2, 19].

Considering the aforementioned challenges, sufficient motivation remains to develop novel methods to improve the performance of multifunctional prosthetic hands. Hence, some assistance studies using multiple-source signals were conducted, and their results demonstrated that it is a possible solution for the problem of insufficient information in the recognition of different types of grasps, particularly for SCIs. A previous study recorded EMG signals from jaw muscle contractions to direct the movement of neuro-prostheses [13]. Recently, a new method combining standard EMG with an inductive tongue control system was implemented to control the five grasp types of a prosthesis [17]. Moreover, other modalities have been proposed to improve the quality of life for disabled people, such as throat microphones, shoulder joysticks, magnetic sensors, and artificial vision [8, 10, 25].

This paper presents a novel control scheme for a two-degree-of-freedom (2-DOF) prosthesis using EMG signals from different facial actions. We hypothesized that the proposed technique can provide an alternative scheme for amputees to control an actual prosthetic device. The rest of the paper is organized as follows. Section 2 addresses the methodology, including the mechanism of EMG responses of facial action and the EMG-control prosthesis system based on a facial-action paradigm. Furthermore, the experimental setups and signal processing method to recognize EMG responses are also described in this section. The experimental results are discussed in section 3. The discussion and conclusion are presented in sections 4 and 5, respectively.

## Materials and methods

The neural pathway mechanism of different muscle responses provides the theoretical foundation for the control of neuro-prosthetic devices. This section first introduces the description of the neural mechanism of facial action. Subsequently, the construction of the EMG prosthesis system based on a facial action control scheme is presented. Finally, the experimental protocol and EMG signal processing methods are discussed, respectively.

### Mechanism of EMG-based facial action and its control model

**Fig. 1:**
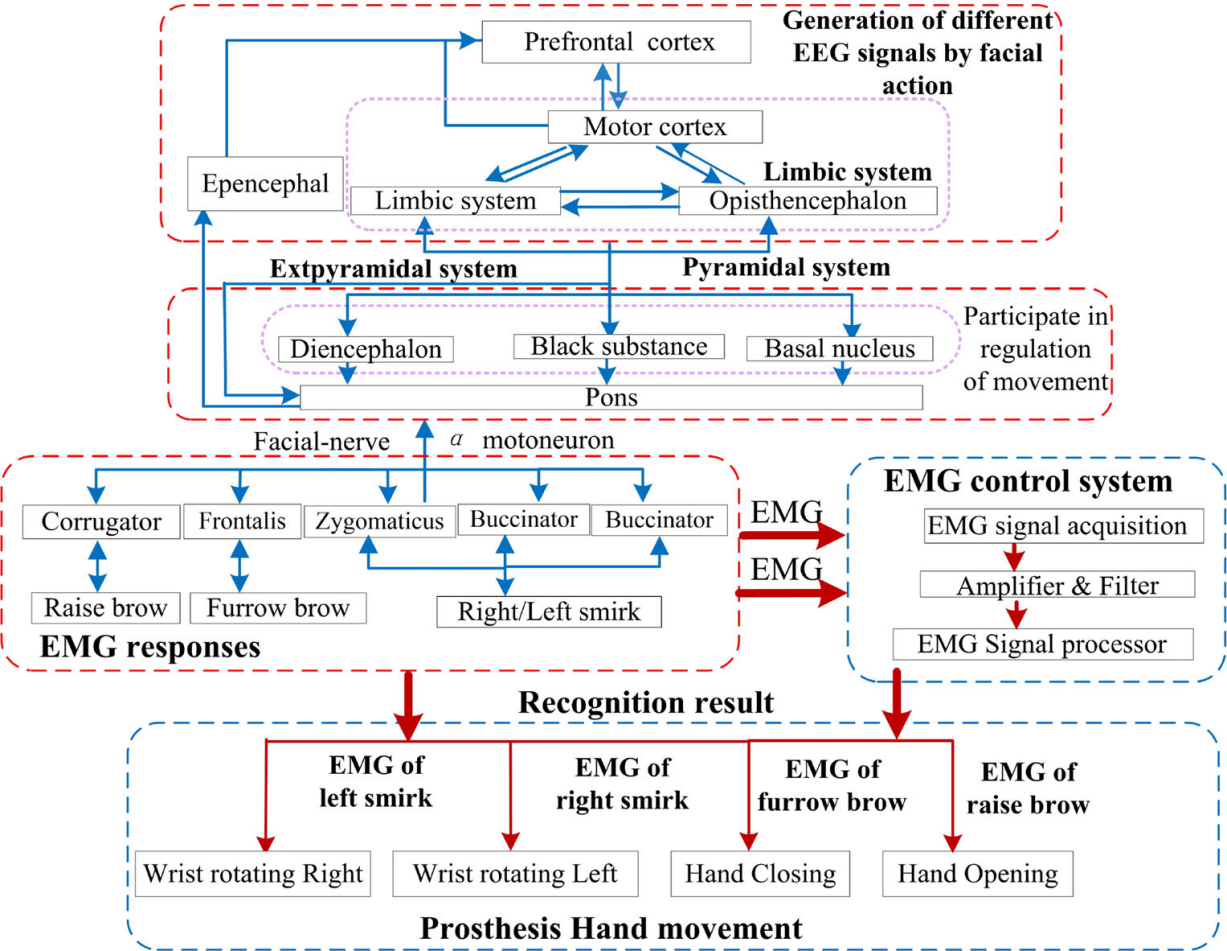
EMG-controlled prosthesis system based on facial action and its schematic description

The research on the relationship between emotional processing and facial action has increased. Previous studies proved that brain activity from the prefrontal and motor cortexes provides a biological foundation to distinguish the movements of facial actions [11, 14, 24, 29, 32, 33]. For expected facial muscle contraction, many factors contribute to the mechanism of a person’s facial action. These include EEG signals response, nervous system transmission, motor neuron generation, and facial nerve transmission [28]. As shown in Fig. 1, information about the anatomy of facial action was examined and a physical model was built to guide the prosthesis-controlling strategy based on the facial-action paradigm.

During the entire facial-action process, the cell column with the same function from the prefrontal and motor cortexes and limbic system are integrated through an inner loop structure; the firing rates of action potentials contain detailed information for the plan and execution of a specific facial action. Part of EEG signals transmits to the low-level central neural system; substance α motor neuron is released by a special spinal motor neuron, which will translate the information to the facial nerve terminals. For EMG delivery and action units, a series of anatomy-based studies on facial action proposed that the movements of muscles during face actions can be divided into two groups with respect to their musculature and unique nerve tracts: upper and lower facial muscle movement in certain combinations [35, 36]. The upper facial muscles control the upper facial movement, which primarily occurs around the brows and eyes. For example, brows can rise from the coordinated contraction of the corrugator, procerus, and orbicularis oculi. The lower facial muscles control the lower facial movement, which primarily occurs around the cheek and mouth. For example, a smile relies heavily on the depressor anguli oris and zygomaticus. Interestingly, researchers observed that electrical muscle activity from facial action can be detected broadly across the scalp and sides of the face because of the extended distribution of facial musculature in those areas [13].

The aforedescribed theory analysis demonstrates that the EMG signals from different facial actions can be distinguished. The characteristics of the facial action mechanism indicated that the signals from facial muscles may eventually have clinical utility. Hence, considering the mechanism of facial action and symmetry of different facial muscle contraction, we constructed the control model for a 2-DOF prosthesis hand. The prosthesis control strategy based on facial action is also introduced in Fig. 1. In this model, a subject was allowed to use four facial actions to complete the grasping exercises of the prosthesis. Table 1 presents the detailed corresponding relationship between the prosthesis movement and facial actions.

**Table 1:** Corresponding relationship between prosthesis movement and facial actions

Hand movement	Hand opening	Hand closing	Wrist rotating right	Wrist rotating left
Facial action	Raising brow	Furrowing brow	Left smirking	Right smirking

### Description of EMG-controlled prosthesis system

According to performance criteria and previous experiences for prosthetic application, the traditional scalp surface recording equipment (Neuracle-W8, Neuracle, China) was used to acquire a subject’s scalp muscle (not brain) activity in this study, which was transformed from facial muscle contractions [16]. In the biological method, the movements of muscles during the actions in the face are measured either by EMG recording or EEG equipment.

**Fig. 2:**
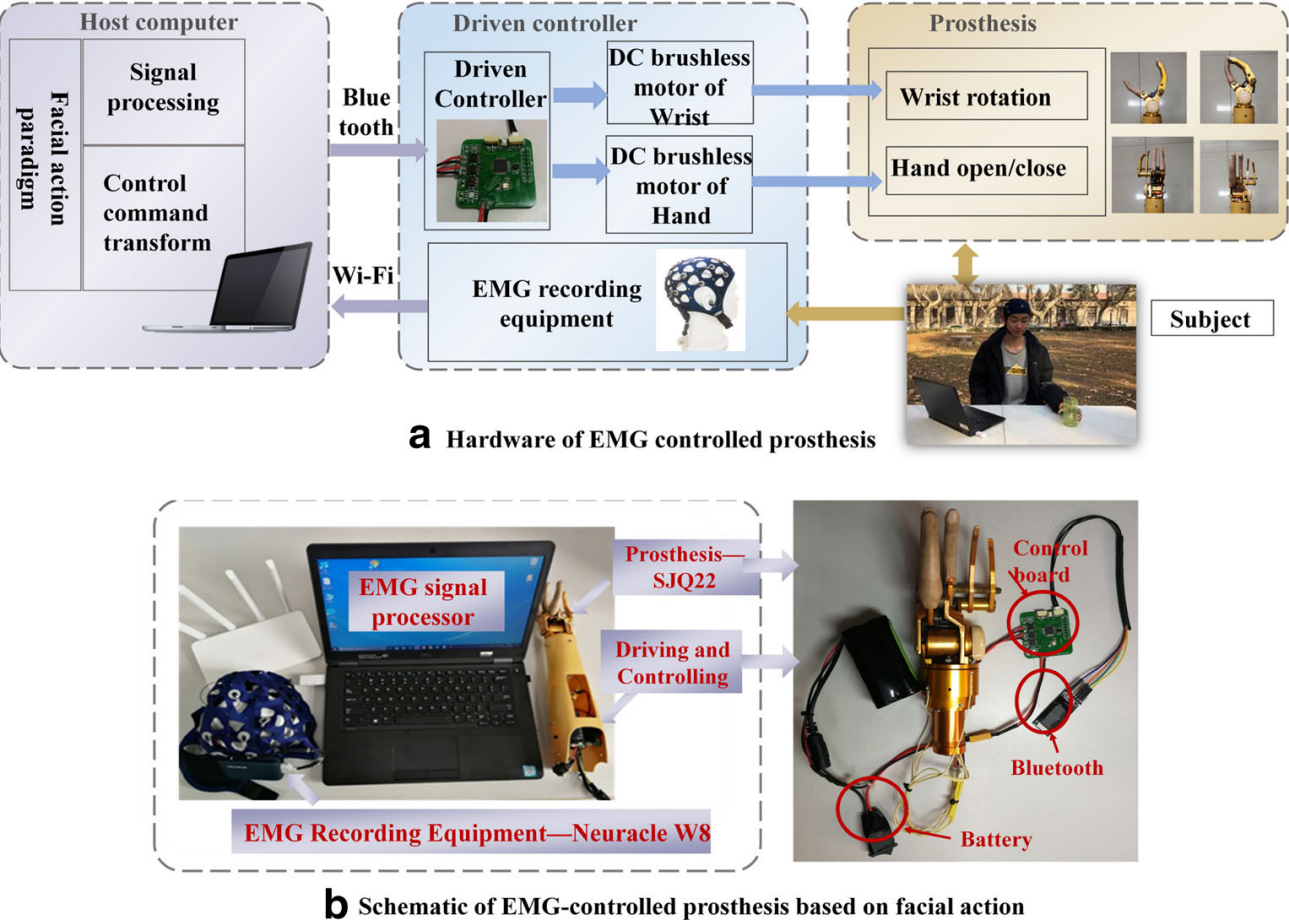
EMG-controlled prosthesis system based on facial action and its schematic description. **a** Hardware of 2-DOF facial EMG-controlled prosthesis system. **b** Schematic of the EMG-controlled prosthesis system based on facial action

The hardware of the EMG-controlled prosthesis system based on facial action is shown in Fig. 2(a) and comprised an EMG data acquisition module, an EMG signal processing module, and a prosthetic module. A microprocessor with an Intel (R) Core (TM) i7-9700 CPU was selected as the EMG signal processing unit. The prosthetic module was composed of a prosthetic hand controller, Bluetooth device, prosthetic hand, etc. In this study, a 2-DOF prosthesis with wrist and finger joints custom-made by Danyang Artificial Limb Co., Ltd. was employed as the control target. The driving and control circuit board was a 4 cm × 4 cm circuit board that we designed, and the main control module selected was an STM32F103C8T6 chip, which satisfied the requirements of the 2-DOF prosthesis control.

The overview of the prosthesis-control strategy is shown in Fig. 2(b). The EMG-controlled scheme enabled a subject to select grasp gestures and directly change the type using different facial actions. When the subject has an intention to operate the prosthesis, the EMG equipment detected the facial EMG contractions and transformed them through Wi-Fi. Subsequently, the EMG processing unit decoded the signals with a corresponding algorithm and a control command was generated to drive and control the unit. Finally, the prosthesis was triggered by the control command and changed its gesture for other facial EMG contractions.

### Subject and data acquisition

A total of nine healthy volunteers between the age of 22 and 30 years old participated in the experiments (7 males and 2 females). All of them had no cognitive deficits. None of them had prior experience with facial action of the EMG paradigm or the proposed experimental procedure. Written informed consent was obtained from each subject before the experiment. The Institutional Review Board of Xi’an Jiaotong University approved the proposed experiment, and all experiments were conducted in accordance with the Declaration of Helsinki.

**Fig. 3:**
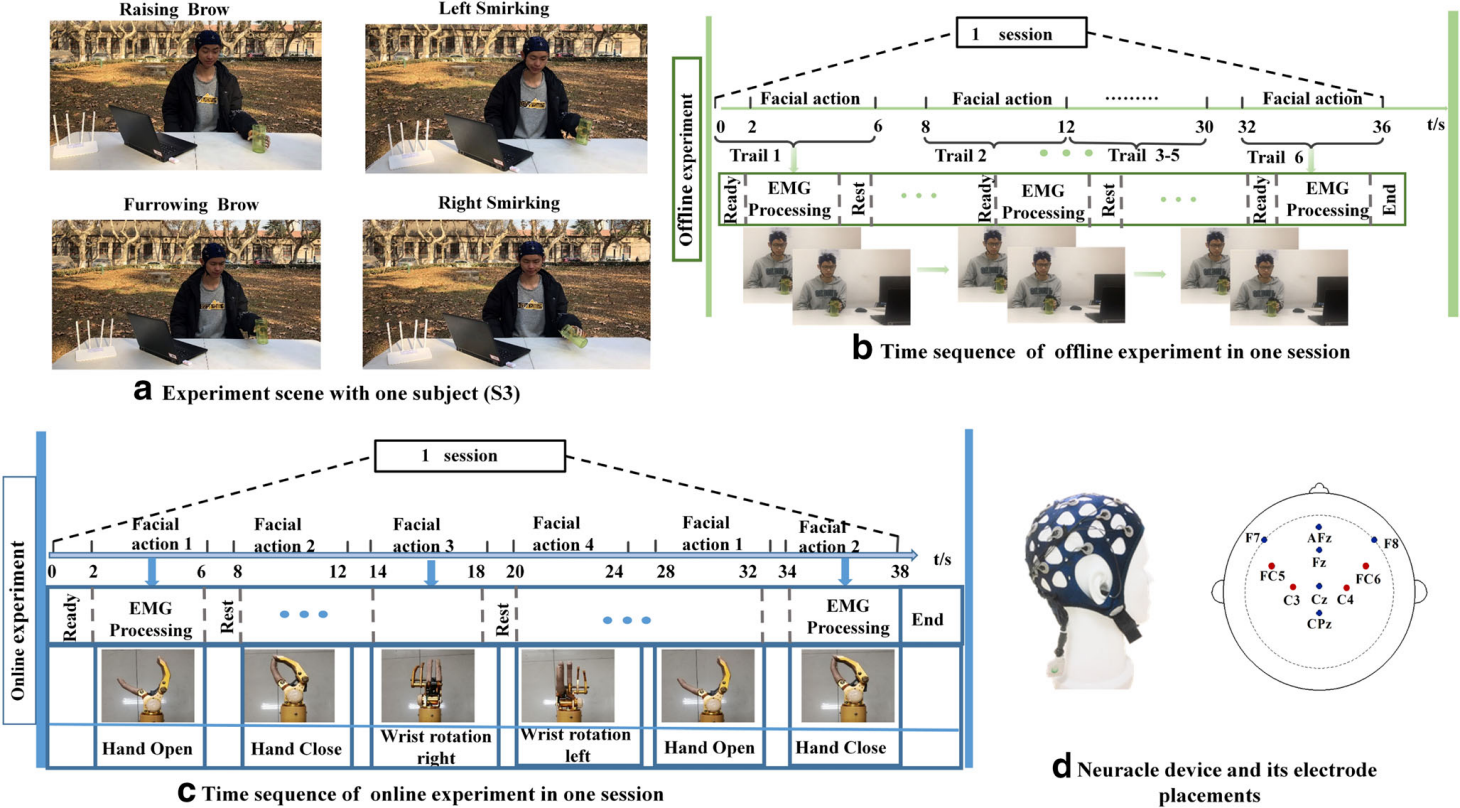
Experimental setup. **a** Experiment scene with one subject (S3) to illustrate the four facial expression tasks used for prosthesis movements. Raising brow corresponds to hand opening, furrowing brow corresponds to hand closing, Left smirking corresponds to wrist rotating to the right, and right smirking corresponds to wrist rotating to the left. Written informed consent for the publication of identifying images was obtained from the subject (S3). **b** Offline experimental time sequence of one session. **c** Online experimental time sequence of one session. **d** Illustration of head amplifier and electrode placements

The previous studies demonstrated that extracting an EMG signal on the scalp using a typical non-invasive BCI was an effective method to record the facial and neck muscle contraction [13]. Surface EMG activity from the movements of muscles during facial actions can be detected broadly across the scalp due to the extended distribution of facial muscles in those areas. Moreover, the facial muscle system is muscular, and the EMG activity is particularly strong near the forehead owing to the underlying facial nerves, which are composed of sensory, motor, and parasympathetic fiber components. Hence, eight electrodes with two references that were set near the facial muscles were used to record EMG signals; the sampling rate was set at 1000 Hz. The electrode setting at AFz was employed as the ground potential, and another electrode setting at CPz was selected as the reference potential. To record robust EMG responses over the scalp, we placed four electrodes on F7, F8, FC5, and FC6 to detect the EMG activity from different facial actions because of their locations near the facial muscles. The electrode description and their placement are shown in Fig. 3(d). The impedances for all electrodes were maintained below 5 kΩ. For lower noise contamination, a Butterworth bandpass filter was applied to the raw signals, and the EMG data in the 50–500 Hz frequency bands were obtained. Furthermore, a notch filter was selected to eliminate power interference.

### Experimental setup

Each subject was asked to complete both offline and online experiments to verify the feasibility of the EMG-controlled method based on facial action. During the experiment, the subjects sat on a comfortable chair stationed 60 cm from the front desk and without body movement. Figure 3(a) depicts the experimental scene from one subject (S3). S3 agreed to have his experiment images published, and a written informed consent was obtained from him.

#### Offline experiment

The offline experiment was conducted in a room. Each subject was asked to repeat four facial actions (raising brow, left smirking, right smirking, and furrowing brow) for ten sessions with a comfortable and consistent level of effort. Every session consisted of six trails. Each trial lasted for 3.5 s including 1 s of preparation, 1.5 s of performing one of four facial actions, and 1 s of a break. The detailed time sequence of the offline experiment is shown in Fig. 3(b). A 5-min rest time was included between every two sessions to avoid mental and muscle fatigue. The indoor experiment focused on investigating the performance of the proposed paradigm, such as recognition accuracy.

#### Online experiment

Because the grasp pattern is one of the most important functions of the human hand movement, the online experiment aimed to imitate drinking water. For the first time, we conducted the online experiment in the outdoors. In the online experiment, four types of hand movements were selected to complete the task of drinking water, which were hand opening, hand closing, and wrist rotating right and left, respectively. The arrangement of the online experiment is shown in Fig. 3(c). During the online experiment, the action sequence was preset and saved on the computer. The subject was asked to perform drinking movements as aforementioned. Each subject was instructed to repeat ten sessions.

Throughout the online experiment, the subjects were instructed to control the 2-DOF prosthesis using facial action tasks, namely, the opening and closing of the prosthesis with the brow actions and rotating the prosthesis wrist with the corresponding mouth actions as detailed in Table 1.

### Methods

**Fig. 4:**
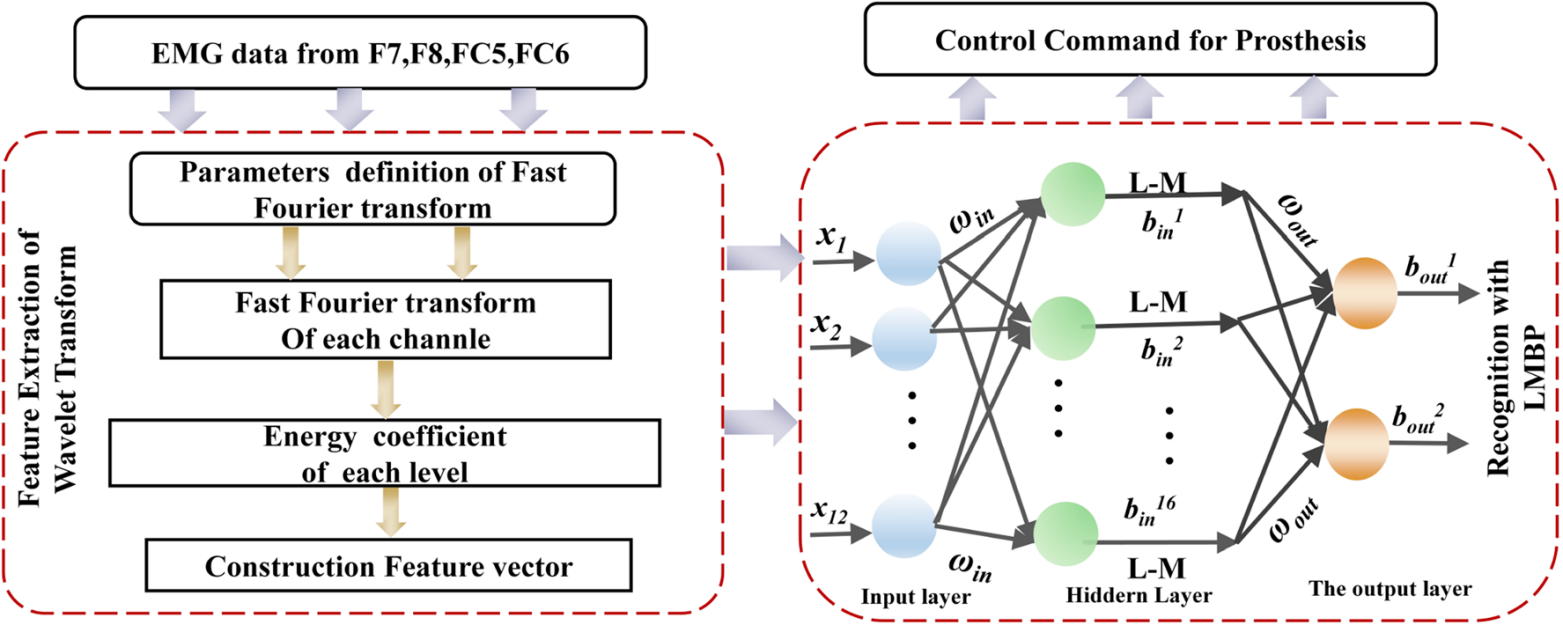
Overview scheme of signal processing method

The fast Fourier transform (FFT) steadily transforms a time-domain signal into different frequency scales. Hence, the FFT was selected to extract EMG features in this study. Furthermore, artificial neural networks (ANNs) have been examined as possible solutions to solving complex problems such as biological signal processing. The back propagation neural network (BPNN) is a hierarchical feed-forward ANN consisting of three or more fully interconnected layers of neurons and is currently the most widely used ANN architecture. The BPNN classifier is well-known to provide good performances when classifying non-linear, self-adaptive, and self-learning sample sets. Thus, BPNN was selected for the EMG signal classification in this study. Figure 4 depicts the entire overview diagram of the signal processing method.

#### Feature extraction

FFT has been widely used in EMG signal processing and is an effective method of extracting the characteristics of random surface EMG data because facial muscle contractions have their unique motivation and spatiotemporal characteristics. Hence, the energy of three frequency bands (64–128 Hz, 128–256 Hz, and 256–512 Hz) from four selected channels (F7, F8, FC5, and FC6) produced by FFT were selected as feature vectors. The statistical parameter of FFT energy coefficients can be obtained using the following equation:

1 $$ {P}_{\mathrm{j}}=\sum \limits_{i=1}^n{y}_i^2\left(j=1,2,3,4\right) $$

where *j* represents the FC5, FC6, F7, and F8 channels, *i* is the FFT coefficient of each sub-rhythm, *n* is the total number of FFT coefficients in each rhythm, and *P*_j_ is the energy of the FFT coefficients of each sub-rhythm from each channel.

Using Eq. (1), features of the three frequency domain ranges (the power of frequency) were extracted on each selected channel. After the feature sets of all channels were concatenated, a 3 × 4 vector of all feature points was provided to the classifier in each facial action trail. Hence, the training database of each subject contained four types of facial actions and each action recorded 6 trails × 10 sessions = 60 trails during the offline experiment. In more detail, the training database of each subject contained 4 × 60 = 240 samples in total. In each sample, a 3 × 4 feature point was computed by the FFT algorithm. The effectiveness of the selected feature extraction method was compared with a wavelet transform (WT) with a mallet algorithm to estimate the effectiveness of the feature extraction of multi-channel EMG data [40]. Based on the frequency band of the EMG signal in 50–500 Hz band, the decomposition of the WT was set at 3, and the wavelet function at Daubanchie 5 (db-5) was adopted to perform the discrete WT.

#### Neural network model based on the Levenberg-Marquardt back propagation algorithm

The pattern recognition of an EMG signal is a method to increase the amount of information gleaned from muscles and eliminate the requirement for isolated EMG signals. In the literature, the majority of studies on different pattern recognition have been used for biological signals [3]. The BPNN has become a popular machine learning model for EMG signal classification owing to its good ability to identify a complex non-linear relationship between input and output datasets. The advantage of this method is that an algorithm for optimal parameter-learning can be used to decrease the mean square errors of performance, which further increases the separability of selected feature vectors [42]. However, the standard BPNN has some defects such as slow convergence rate and easily getting into local dinky value. The performance of the BPNN is highly dependent on its optimal parameter values, which are calculated by a parameter-learning algorithm [4, 5]. Hence, the novel optimization algorithm by Levenberg-Marquardt used in a majority of pattern recognition studies, which is derived from the steepest-descent method in combination with Gauss-Newton methods for network weights and the optimization of bias variables [26], was used in this study.

The design of the BPNN for the EMG recognition system involved three factors: network design, training datasets, and learning algorithms.

For the network structure, the input layer depended on the result of feature extraction and the output layer denoted by the number of control types. Because the input feature set of each trial from each subject with the energy of three frequency bands (64–128 Hz, 128–256 Hz, and 256–512 Hz) from four selected channels, the input vector *x* = [*x*_*1*_, *x*_*2*_, …, *x*_*12*_] of each sample, the corresponding input layer of the BPNN had 12 nodes. In this study, the number of classes in the training datasets was four types of EMG signals from different facial actions for prosthesis control. For a binary system classification, the predicted label value *y* from 2 output nodes can differentiate the input feature vector *x*, where *y* = [*y*_*1*_, *y*_*2*_] and *y*_*i*_ ϵ (− 1, 1). In more detail, the output vectors *y* = [0, 0], [0, 1], [1, 0], and [1, 1] represented the furrowing brow, raising brow, left smirking, and right smirking classes, respectively. Hence, the output layer of the proposed LMBP model had 2 nodes. The hidden numbers of neurons have a significant effect on the generation of the training mode, hence, selecting the appropriate value in the course of the experiment is very important. Referencing the empirical rules of hidden node description presented by Adamowski J [1], the range of the number of hidden nodes was obtained. Then, the exhaustive search strategy was used to find the optimal value of the number of hidden nodes based on the better performance of proposed LMBP. After the experiment’s repeated results, the number of hidden neurons in the hidden layer that must be optimized through the error procedure is defined as 16. Hence, the structure of the LMBP model used in this study consisted of an input layer with 12 neurons in a single hidden layer composed of 16 neurons, and an output layer consisting of 2 neurons denoting the different facial actions.

##### Preparing the training and test data for the LMBP classifier

To detect the robustness of the proposed BPNN and prevent an over-fitting problem, we used a ten-fold cross-validation to investigate the classification accuracy: the input vectors and target vectors were randomly divided into ten sets, and the cross-validation was repeated four times. During each validation, nine subsets of data were used for training and one for testing. During the experiment, each subject’s data were used to train his or her own classifier.

Parameter optimization has a significant effect on the classification performance of BPNN. In this study, the adaptive gradient search strategy combined with Gauss-Newton searched strategy, which is called Levenberg-Marquardt optimization or LMBP algorithm, was used to optimize the supervised model parameters. The Levenberg-Marquardt optimization algorithm has two advantages. First, it retains the local optimal characteristics of Newton’s method and has the global benefits of the gradient method, which speeds up the convergence of the BPNN. Second, this algorithm exhibits global convergence, a guaranteed rate of local convergence for both zero and non-zero small residual problems, and it decreases the time complexity of the predictive model compared with the traditional BPNN parameter adjustment method [22]. Hence, the LMBP neural network model served as a recognition approach to identify the accuracy of the prosthesis gestures in this research.

The basic element of an ANN is the neuron, which is a logical mathematical model that simulates the behavior and functions of a biological neuron [26]. In the LMBP-optimization algorithm, the log-sigmoid activation is adopted to compute the forward output of neurons, which can be computed by:

2 $$ f\left({s}_j\right)=\frac{1}{1+\exp \left(-\sum \limits_{i=1}^m{w}_{lj}{x}_i+{\theta}_j\right)} $$

where *ω*_*lj*_ is the connection weight from the *l* to the *l* + 1 layer, *θ*_*j*_ is the bias value for the *j*th hidden node in *l* + 1, and *j* is the number of nodes in the hidden layer.

In LMBP, the mean square error is the key aspect affecting the performance of network; it can be given by the following expression:

3 $$ E(Q)=\frac{1}{2}\sum \limits_{m=1}^M{\left\Vert {P}_m-{Y}_m\right\Vert}^2=\frac{1}{2}\sum \limits_{m=1}^M{e}_m^2(Q) $$

where *M* is the number of input features and *e*_*m*_ (*Q*) is the error.

The gradient of the Jacobian matrix and Hessian matrices in the LMBP algorithm are:

4 $$ {\displaystyle \begin{array}{c}\nabla E(Q)={J}^T(Q)e(Q)\\ {}H(E)={J}^T(Q)J(Q)+K(Q)\end{array}} $$

where *e* is the unit matrix and *J*(*Q*) is the Jacobian matrix.

Subsequently, the correction *∆Q*_*k*_ of each training can be given by:

5 $$ \varDelta {Q}^k=-{\left[{J}^T(Q)J(Q)+\mu E\right]}^{-1}{J}^T(Q)e(Q) $$

When seeking the optimal-parameter solution of a function, the LMBP algorithm exhibits the ability of fast local convergence of the Gauss-Newton method. In addition, if it diverts from the optimal values, the algorithm has characteristics of a global search strategy with the gradient-descent method. This ensures that each update of the weight and bias value decreased the error and avoids network fluctuations.

**Fig. 5:**
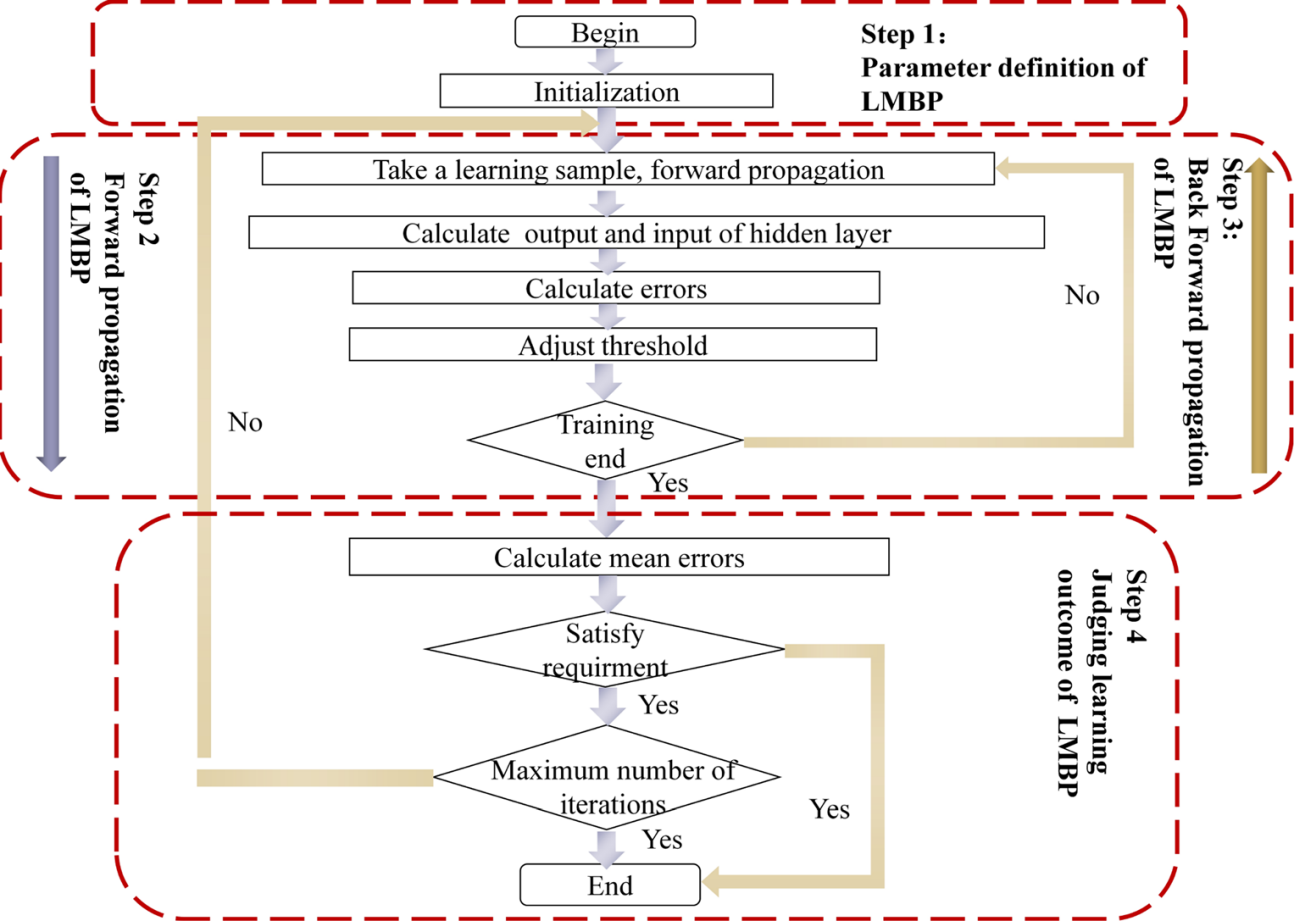
Scheme of classifier training for the LMBP-optimization algorithm

In Eq. (5), if the correction satisfies the condition *∆Q*^*k*^ < *ε*, the training is ended; otherwise, it is continued. After the parameters and training are selected, the LMBP classifier is generated. The flow of the LMBP algorithm is shown in Fig. 5.

The steps to realize the LMBP algorithm are as follows:

1. Initialize parameters, including network weight, learning rate, and threshold error. Set iterations and total error to zero.
2. Collect the input data and feed it to the input layer units.
3. Calculate the outputs of hidden layer units using LMBP optimization.
4. Calculate the outputs of output layer units using LMBP optimization.
5. Calculate the error and total error.
6. Calculate the output layer units, and adjust the weights between the output and hidden layer units.
7. Calculate the hidden layer units, and adjust the weights between the hidden and input layer units.
8. If there are more feature vectors in the file, go to step 4.
9. If threshold error ≥ total error, stop; else, go to step 3.

The parameter-optimization method was compared with the Adam algorithm to estimate the effectiveness of the Levenberg-Marquardt optimization in the recognition of multi-channel EMG data.

#### Statistical analysis

Before statistically comparing classification accuracy between two methods (WT vs FFT), data were statistically tested for normal distribution (one-sample Kolmogorov Smirnov test) and sphericity (Mauchly’s test). A post hoc comparison was performed using Tuckey–Kramer tests. A significant analysis is generally based on the hypothesis testing of normal distributions. A student’s paired *t* test method was applied to assess the differences in the recognition accuracy of four facial actions. The one-way ANOVA was applied to assess differences in the energy coefficients among four facial actions. The Greenhouse–Geisser correction was applied for *p* value adjustments.

## Experimental results

Before addressing whether our EMG-based facial action strategy could be used to control a 2-DOF prosthesis outdoor, the feasibility of the proposed paradigm required to be thoroughly validated through the offline experiment. Subsequently, the online experiment was conducted to validate the effectiveness of our proposed system to control prostheses.

### Offline analysis

**Fig. 6:**
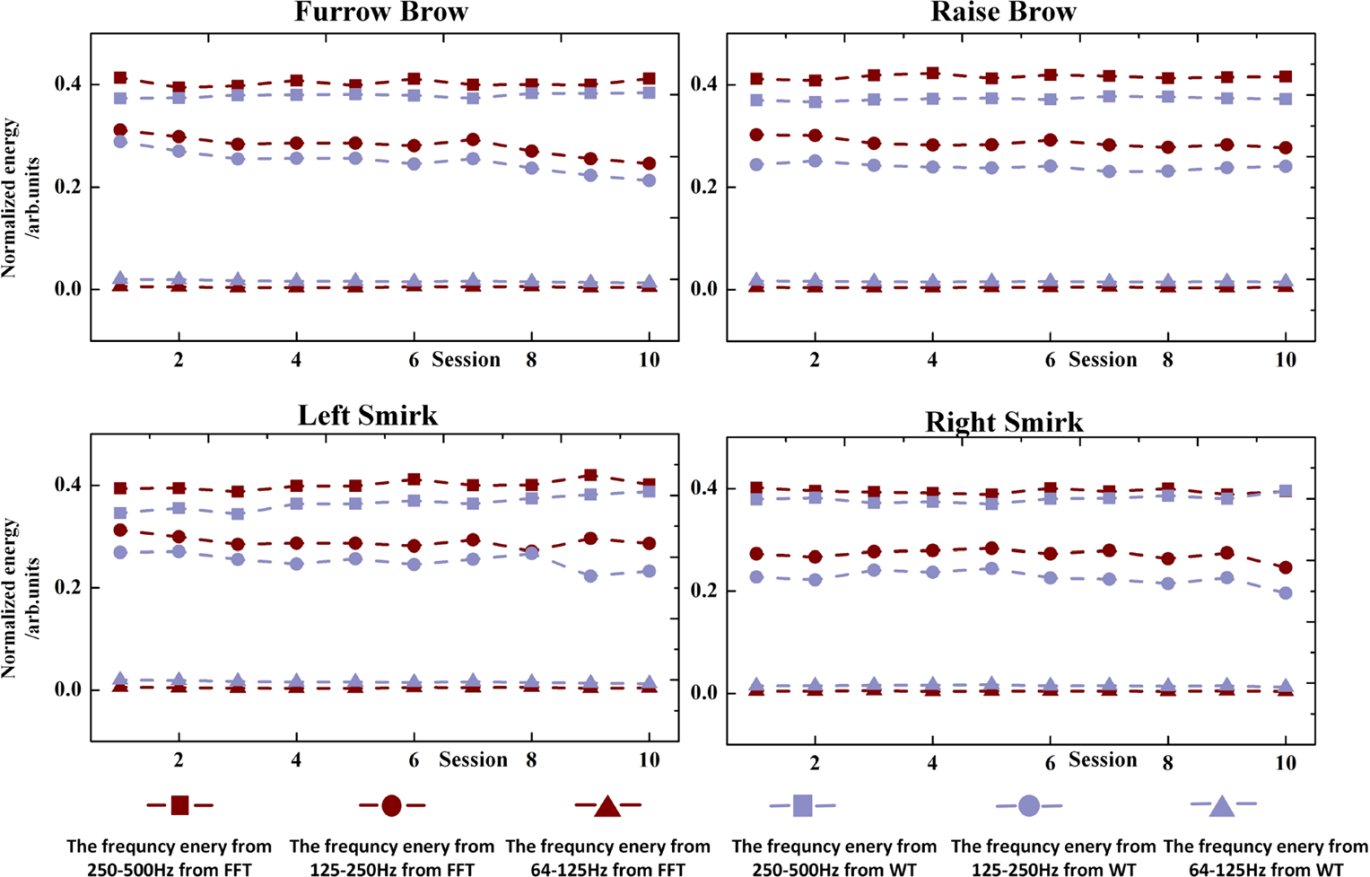
Feature sets of four facial actions from prominent subject (S6)

To demonstrate the effectiveness of the proposed facial EMG-controlled system, we compared the averaged frequency domain and time-frequency domain features from one prominent subject (S6). Other subjects indicated similar results. Figure 6 depicts the results of the calculation of both the energy coefficients from WT and FFT using the averaged data from F7, F8, FC5, and FC6. The detailed information of each session for S6 was plotted, and all facial actions were investigated. We observed that the difference in feature sets from the FFT was more remarkable than the feature sets from the WT in the same facial action. Furthermore, the EMG activities of four facial actions indicated significant differences in characteristics of selected feature sets of each action and all offline experiments indicated similar performances.

**Fig. 7:**
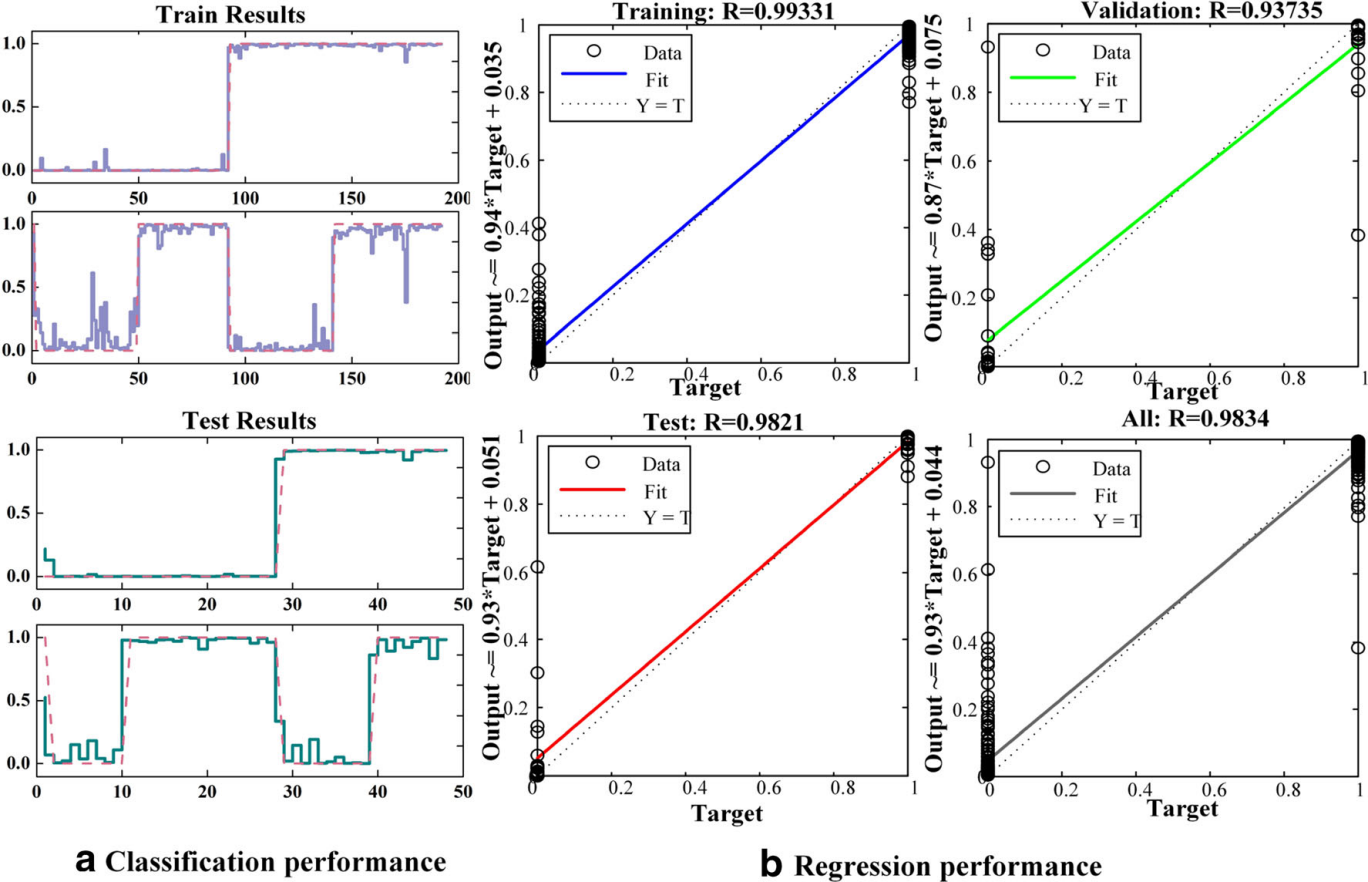
Performance of the LMBP model during training and validation for S6. **a** Classification performance. **b** Regression performance

The performance of the LMBP classifier was investigated to better understand the effectiveness of the selected recognition algorithm. Figure 7(a) depicts the performances of the LMBP classifier in the training and validation stages from S6. During the offline training stage, the validation performance attained its maximum at 12 epochs and the root mean squared error between the output and the predicted targets varied slightly. Moreover, the linear regression performance of the trained model is shown in Fig. 7b; the training and test regression results were 0.99331 and 0.93735, respectively.

The experimental results indicated that the LMBP model with 16 hidden neurons could accurately estimate the type of facial action through selected features. Furthermore, the performance obtained through the aforedescribed spatiotemporal analysis also demonstrated that the FFT combined with LMBP was an efficient algorithm to distinguish different facial actions, and the selected FFT coefficients were the more effective features to describe the characteristics of facial action.

Table 2 presents the comparison results of all subjects and conditions; the averaged accuracies of WT and FFT were 83.05 ± 5.67% and 95.45 ± 3.10%, respectively. The paired sample *t* test was used to test the performance of four facial actions, and the analysis results indicated that the accuracy of FFT methods increased significantly compared with WT. In the EMG-based facial action paradigm, all accuracies were higher than 73.47% during the period. For the EMG-based facial action paradigm with FFT, the highest recognition accuracy was obtained by S7, which was up was 98.89 ± 1.33%; the lowest accuracy was 91.11 ± 4.95% for S9. In particular, the accuracies from three subjects (S2, S6, and S7) were significantly higher than 97%. All the subjects indicated similar results in the proposed paradigm. However, despite these general experimental results, some inter-subject variability occurred. This phenomenon may have been caused by attention attenuation or mental fatigue for the facial repetitive tasks.

**Table 2:** Offline accuracies of each subject in facial actions with two feature extraction methods

	Accuracy (%)	
	W T	FFT
Subject 1	87.36 ± 5.43	96.25 ± 3.94
Subject 2	89.13 ± 5.69	97.92 ± 2.36
Subject 3	78.19 ± 5.33	96.81 ± 2.60
Subject 4	82.64 ± 5.93	93.33 ± 3.07
Subject 5	73.47 ± 6.75	95.83 ± 3.52
Subject 6	78.75 ± 6.92	97.50 ± 1.96
Subject 7	91.53 ± 4.88	98. 89 ± 1.33
Subject 8	90.28 ± 6.23	94.83 ± 4.24
Subject 9	76.11 ± 3.94	91.11 ± 4.95
Avg ± Std	83.05 ± 5.67	95.45 ± 3.10

To determine the efficiency of the selected parameter-optimization method in the EMG-controlled system based on facial action, we estimated the offline classification accuracy using two different optimization methods. As shown in Table 3, the grand average offline accuracy obtained for all subjects was higher than 84.87 ± 14.94%. The performance of the proposed optimization algorithm indicated a higher classification rate and better robustness for all subjects. Statistical analysis was used to assess the performance under two conditions. Significant differences were observed among the two conditions using a student’s paired *t* test (*p* < 0.05). The experimental results validated the efficiency of the proposed method in detecting the characteristics of EMG signals from different facial actions.

**Table 3:** Offline accuracies of each subject in facial action with two parameter-optimization methods

	Accuracy (%)	
	LMBP	Adam
Subject 1	96.25 ± 3.94	85.88 ± 15.66
Subject 2	97.92 ± 2.36	87.00 ± 10.16
Subject 3	96.81 ± 2.60	87.17 ± 14.04
Subject 4	93.33 ± 3.07	83.83 ± 13.88
Subject 5	95.83 ± 3.52	80.83 ± 14.81
Subject 6	97.50 ± 1.96	85.75 ± 12.63
Subject 7	98. 89 ± 1.33	88.78 ± 16.31
Subject 8	94.83 ± 4.24	85.00 ± 19.94
Subject 9	91.11 ± 4.95	79.58 ± 17.03
Avg ± Std	95.45 ± 3.10	84.87 ± 14.94

Overall, the offline analysis result proved that the proposed system indicated good performance and could be further used in practical applications.

### Online analysis

The aforedescribed results demonstrated the feasibility of the proposed EMG control method based on facial actions. Hence, the online experiments focused on the practical performance of our proposed system. An EMG-controlled prosthesis based on facial action was used to imitate the normal daily tasks such as water drinking.

**Fig. 8:**
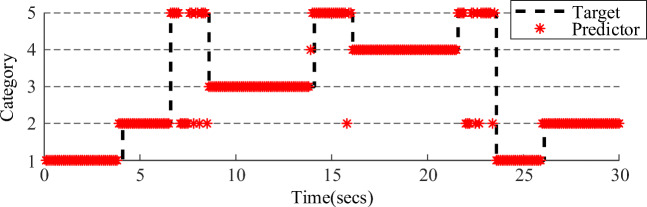
Best recognition performance within a single session for S7

The online task asked subjects using the 2-DOF prosthesis to complete a water-drinking process with four facial actions. The subjects had to produce different control commands to operate one complete drinking cycle with four discrete prosthetic gestures. In the prosthesis operating stage, subjects were required to hold on one same facial action before a prosthesis movement decision was generated at the end of 1.5 s. Moreover, the prosthesis would remain in the previous gesture before any new control commands were generated. In the online experiment, each subject had his or her own classifier, and all the offline data were applied to train the LMBP classifier. The best recognition performance within a single session for S7 is shown in Fig. 8, which further demonstrated the feasibility of the EMG based facial action paradigm for prosthesis control.

Table 4 summarizes the recognition results from all subjects across all sessions. It indicated that the averaged accuracy was 95.39 ± 3.13% from nine subjects across all sessions. The highest and lowest ones were 100.00 ± 0 and 86.78 ± 10.31%for S7 and S9, respectively. Furthermore, the classification error rate was less than 10.31% on all subjects, and the mean value was 3.13%. The results indicated that these four facial actions could be classified by an LMBP classifier even if the prosthesis hand was moving.

**Table 4:** Online accuracies of each subject in different facial actions

	Subject 1	Subject 2	Subject 3	Subject 4	Subject 5	Subject 6	Subject 7	Subject 8	Subject 9	Avg
Acc (%)	96.67	96.67	95.00	98.33	96.67	98.33	100	90.11	86.78	95.39
Std (%)	7.03	10.19	7.03	5.27	7.03	5.27	0	8.61	10.31	3.13

**Fig. 9:**
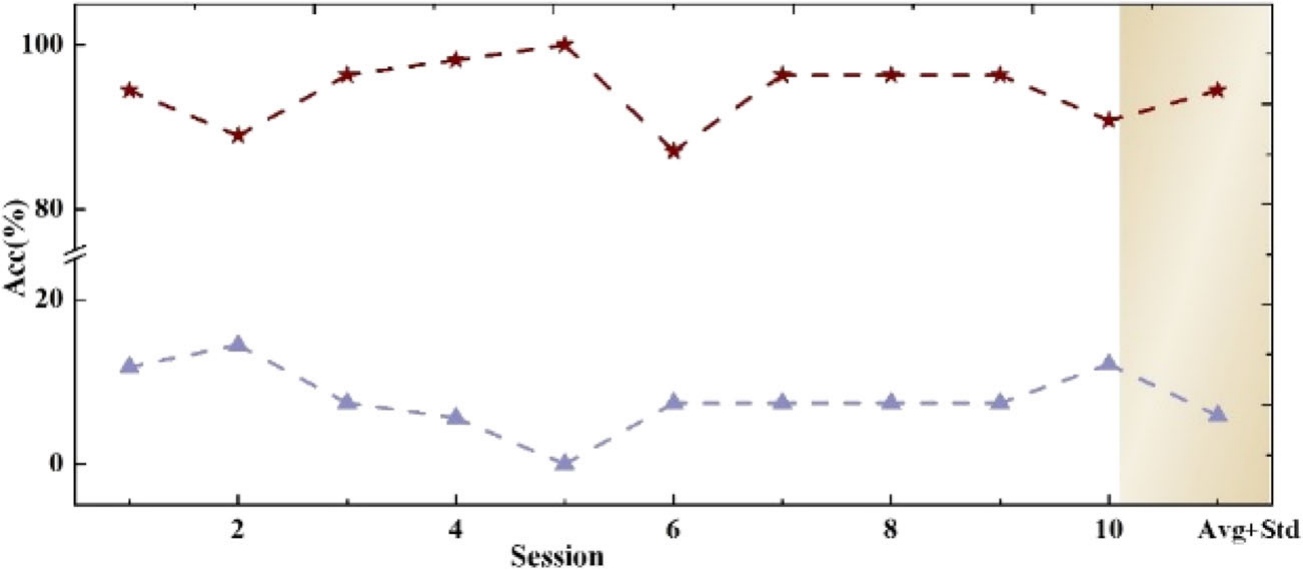
Prosthesis-controlling accuracies and standard deviations of each session

To access the tendency of mental fatigue during all the online tasks, we calculated the accuracy of each session for all subjects in Fig. 9. No significant decrease in accuracy was observed over the entire experiment. A one-way ANOVA analysis for the session’s performance was conducted to further analyze its feasibility. No significant difference was observed in the accuracies from nine subjects during each session (*p* > 0.05). Overall, all the results demonstrated the efficiency of the proposed system, which can be further applied to prostheses control.

## Discussion

In this study, a novel EMG control scheme based on different facial actions was proposed, and its efficiency and feasibility applied in operating a 2-DOF prosthesis were assessed. Four facial actions with distinctive facial muscle movements were selected to generate different EMG signals, which were further used to control peripheral devices. Both offline and online experiments were conducted, and the experimental results demonstrated that the EMG-control prosthesis based on a facial action system can be considered an alternative prosthesis control scheme with good performance and feasibility. Compared with existing studies, the superior performance obtained by the proposed method can be illustrated by the following aspects.

### Mechanism of EMG-based facial action

The mechanism of the EMG-based facial action and its control model was analyzed from the brain responses of the cortexes particular to facial muscle contractions. Our previous study demonstrated that the prefrontal, motor, and limbic cortexes have a fundamental function in the completion of different facial actions [24]. Hence, the bio-signals from different facial muscles contain abundant and sophisticated information during their actions. The mechanism of people’s facial actions is composed of the EEG signal generation, nerve system transmission, motor neuron generation, facial nerve transmission, and eventually the realization of facial muscle contractions. Owing to the different involvements of muscle segments for different facial actions, the face can simply be divided into upper, lower, left, and right face [36]. Notably, the EMG signals from different facial actions can be fully decomposed. Although the EMG response of facial action cannot be limited to the proposed system, the mechanism of EMG responses of facial action and its control model has its unique value in the representation of facial action from various aspects. Hence, using a physical model to analyze the facial-muscle-contraction mechanism during the specific task provides a further method to predict the feasibility of a novel EMG-controlled system based on a facial action paradigm.

### Performance of the proposed EMG control system based on facial action

Unlike widely used EMG-decoding methods, a traditional FFT with LMBP algorithm was proposed in this study for feature extraction and pattern recognition to analyze the EMG data from different facial actions. During each action, the EMG signal was separated into three frequency bands to investigate the detailed frequency characteristics. For the same set of subjects, the features from the FFT algorithm outperformed those from the WT in every scenario of the proposed paradigm. Englehart [18] investigated three feature extraction methods, (WT, wavelet transform packed, and FFT). He demonstrated that using a wavelet-based feature set exhibited better performance than others in traditional EMG control prosthesis methods. Time-frequency analysis methods (wavelet-based methods) are known to be more complex than the frequency methods. In our proposed paradigm, the data were essentially stationary in every analysis window; thus, the feature set was computed using a simple method. For the same reason, there is no advantage in using time-frequency methods such as the wavelet packet feature set, which was demonstrated to be significantly effective in the classification of transient signals.

Furthermore, a robust classifier based on LMBP was constructed, which produced good classification performance on both offline and online experiments, with averaging accuracy values in 95.45 ± 3.10% and 95.39 ± 3.13%, respectively. In particular, the highest and lowest accuracies were 100% and 86.78 ± 10.31% from S7 and S9, respectively, during the control of the 2-DOF prosthesis. These results theoretically indicated that the EMG-controlled prosthesis based on facial action has the potential for practical applications.

### Comparison with other methods

As aforedescribed, most previous EMG control prosthesis used surface EMG signals from residual muscles after amputations, and their application focused on static inter-scenarios. In the work of Nasser et al., an anatomical shoulder and prosthetic elbow joint were enabled with simultaneous movement via EMG signals [6]. Michele et al. succeeded in controlling precise artifact hand movements using surface EMG signals, and demonstrated an accuracy of 91% [7]. However, these types of EMG-controlled prostheses highly relied on residual muscle conditions, which, particularly for amputations, cannot provide sufficient myoelectric signals. Moreover, most of these studies focused on a fixed working environment.

To address this deficiency, this study proposed a novel prosthesis control strategy based on facial actions, and all data collection did not rely on whether participants were amputated or not. Furthermore, the purpose of this paradigm focused on multiple outdoor scenarios when undergoing activities of daily life in addition to the classification performance. Its flexibility, stability, and the effect of mobility had slight effects on the performance of the classification. Participants were provided more opportunity to restore some degree of functional ability to interact with their surroundings, particularly for some severely paralyzed patients.

### Limitations and further work

All the aforedescribed experimental results demonstrate the advantages of the proposed method; however, there still have some limitations should be considered. In this study, the results were only evaluated from healthy subjects and individual variation was not considered. In the future, more subjects should be involved in the EMG-controlled prosthesis method based on facial action, particularly for disabled people. Another limitation is that only four facial actions were selected in this study. In a further study, more facial action will be considered to realize more precise prosthesis action. In addition, alternative methods of EMG decoding such as deep learning neural networks will be employed to investigate whether they can aid in decreasing the effect of individual variation and be more applicable to amputee subjects. Moreover, some optimization algorithms of hyper-parameters [4] will be utilized to optimize the ANN model to obtain better classification accuracy, such as genetic optimization based methods .

## Conclusion

In this study, a novel prosthesis control method based on surface EMG signals from different facial actions was proposed. Compared with traditional EMG control methods, a significant improvement was achieved in the proposed method on all the subjects. By using FFT combined with LMBP algorithm, the averaging classification accuracies were 95.45 ± 3.10% and 95.39 ± 3.13% in the offline and online experiments, respectively. The results of this study might be useful in realizing the control of multifunctional myoelectric prostheses for disabled people.
